# Pomegranate (Punica granatum L): A Fruitful Fountain of Remedial Potential

**DOI:** 10.7759/cureus.45677

**Published:** 2023-09-21

**Authors:** Kalyani R Kshirsagar, Swanand S Pathak, Sejal M Patil

**Affiliations:** 1 Clinical Research, School of Allied Health Sciences, Datta Meghe Institute of Higher Education and Research, Wardha, IND; 2 Pharmacology, Jawaharlal Nehru Medical College, Datta Meghe Institute of Higher Education and Research, Wardha, IND; 3 Clinical Research, School of Allied Health sciences, Datta Meghe Institute of Higher Education and Research, Wardha, IND

**Keywords:** flavones, anthocyanins, health restoration, nutritional benefits, ellagitannins, punica granatum

## Abstract

Pomegranate (Punica granatum L.) has long been used for medical purposes. Punica protopunicas and Punica granatum L. are two prominent species of pomegranate, generally known as "Anar" and farmed worldwide. Its medicinal value is documented in several ancient texts. This review article aims to provide a comprehensive overview of the remedial uses of pomegranate in traditional and modern medicine. The methodology employed for this review involves a systematic literature search, collection of relevant articles, and critical analysis of their content. The review covers the botanical properties, phytochemical composition, and diverse remedial applications of pomegranate, including its antioxidant, anti-inflammatory, anticancer, cardiovascular, antimicrobial, and dermatological properties. The gathered data emphasizes the potential benefits of pomegranate-derived compounds in managing a range of health issues. This review sheds light on the importance of pomegranate as a valuable natural resource for various therapeutic interventions and encourages further research to unlock its full remedial potential. Traditional medicine is gaining popularity to restore health to individuals with few negative effects. Due to the existence of key phytochemical elements such as flavonoids, punic acid, ellagic acid, anthocyanins, ellagitannins, flavones, and estrogenic flavonoids, it has a wide range of clinical applications.

## Introduction and background

Punica granatum L. has been used in holistic medicine for a long time. Pomegranate (Punica granatum L.) is a significant fruit crop that can thrive across diverse agro-climatic conditions. This fruit is a spherical grenade, encompassing numerous arils filled with deep red, juicy content. These arils are contained within a shiny, resilient pericarp, or peel, and are topped with a lasting calyx [1]. The P. granatum fruit contains remedial properties, including antibacterial and anti-inflammatory properties. Skin and breast cancer are both inhibited by pomegranate oil. It is high in antioxidant activity, while seed oil contains phyto-estrogenic chemicals. Pomegranate bark and fruit treat dysentery, diarrhea, and intestinal parasites. Seeds are used as a tonic for throat infections and heart diseases. It is used to treat hemorrhoids and to clear the nose and gums [2].

Geographical distribution

One of the earliest frequent master fruits to humans was the pomegranate, Punica granatum L. It is found in Central Asia, the Himalayas, the Mediterranean, and the Middle East. It grows well in California and Arizona and in South Asia, the Mediterranean, and the Middle East [3]. Table 1 contains the taxonomical classification of Punica granatum L [4].

**Table 1:** Taxonomic Classification of Punica granatum [4]

Taxonomic Rank	Taxon
Kingdom	Plantae
Division	Tracheophyta
Class	Magnoliopsida
Order	Myrtales
Family	Lythraceae
Genus	Punica
Species	Granatum
Common name	Pomegranate/ Anar

## Review

Methodology

**Figure 1:**
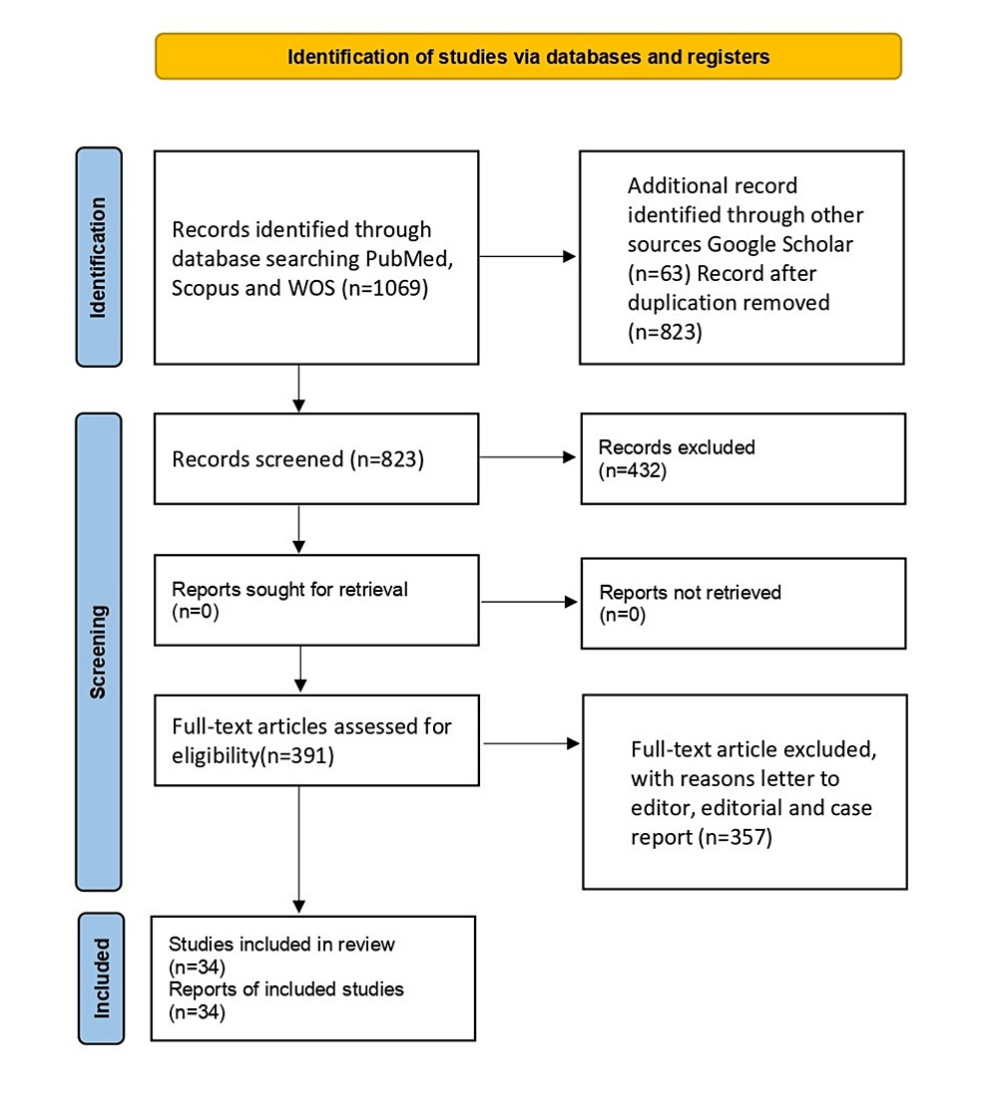
Identification of studies via database and registers

The methodology for this review involved a systematic literature search across databases such as Scopus, Google Scholar, PubMed, and Web of Science. Keywords such as "Punica granatum," "pomegranate," "phytochemicals," and "remedial uses" were used in various combinations to identify relevant articles. Peer-reviewed research articles, review papers, and books published within the last two decades were considered for inclusion. The article deduction flow chart is depicted in Figure 1. 

Nutritional information

The fruit's edible component, particularly the arils, is essential and nutritious and rich in phenolic, inedible fragments of the peel contain high amounts of even essential and biologically active nutrients compared to edible arils. Pomegranate peel is rich in vitamins, minerals, live acids, dietary fiber, and phenolic substances including proanthocyanidin, ellagitannins, and gallotannins in hydrolyzable tannins and precipitates tannins such as flavonoids and flavanols [5].

Phytochemistry

Several parts of the punica granatum, such as the seeds, peels, fruit, and leaves, have diverse chemical components. Flavonoids, flavanols, anthocyanins, and flavan-3-ol are the most common flavonoid components found in pomegranate peel. It also contains ellagic acid derivatives, precursors, and up to 0.2 percent ellagic acid [6].

Flavonoids

Flavonoids are a class of compounds found in pomegranates, which includes flavonols such as luteolin, quercetin, and kaempferol present in the peel extract. Additionally, anthocyanins are another group of compounds found in pomegranates, primarily located in the arils. Anthocyanins are responsible for the vibrant red color of pomegranate juice (PJ). Notable pomegranate anthocyanins include pelargonidin-3-glucoside, cyanidin-3-glucoside, delphinidin-3-glucoside, pelargonidin 3,5-diglucoside, cyanidin 3,5-diglucoside, and delphinidin 3,5-diglucoside [7]. Flavonoids are composed of phenolic substances. It is located in the roots of the plant. Due to the presence of flavonoids, the roots have antioxidant, anti-inflammatory, anti-viral, and anti-microbial properties. Because of its ability to scavenge free radicals and prevent their formation, it possesses antioxidant qualities [8].

Punicic Acid

Punicic acid and its conjugates, which are crucial elements of the pomegranate's hydrophilic fraction (Punica granatum L.) breast cancer cell lines are resistant to the inflammatory effects of seed oil (80% aqueous methanol extract). Amazingly, it shows that two breast cell lines' cell viability is declining [9]. Numerous qualities, such as antidiabetic, antiobesity, and antiproliferative action, are among its many attributes [10]. Physiological disorders such as atherosclerosis, rheumatoid arthritis, metabolic syndrome, and inflammatory bowel disease, are prevalent worldwide. They share a common hallmark characterized by the presence of highly activated inflammatory cells, including macrophages, neutrophils, and monocytes, as well as an overproduction of pro-inflammatory mediators and reactive oxygen species (ROS). These factors contribute to tissue damage and exacerbate the inflammatory response associated with these conditions. One promising avenue for managing these disorders is through the use of natural dietary supplements. Among them, PA (PSO), or polyunsaturated fatty acids, has emerged as a particularly effective option. PA exhibits notable efficacy in countering ROS and myeloperoxidase (MPO)-mediated tissue damage, which is a critical aspect of the inflammatory cascade in these diseases. By incorporating PA into one's dietary regimen, it becomes possible to dampen the excessive activation of neutrophils and help recalibrate the immune response. This not only alleviates the symptoms but also aids in slowing down the progression of these disorders. Additionally, PA's antioxidant properties play a pivotal role in mitigating the damage caused by ROS, further contributing to the overall management of these conditions. In summary, the pathophysiology of common physiological disorders characterized by rampant inflammation and immune cell activation can be effectively addressed through natural dietary supplements like PA (PSO). These supplements offer a multifaceted approach by curbing ROS, MPO-mediated damage, and excessive neutrophil activation, ultimately providing relief and control over these chronic conditions [11].

Ellagic Acid

Ellagic acid (EA) exhibits multiple biological activities. EA shows antihepatotoxic, anticholestatic, and antihepatocarcinogenic activities that enhance the hepatic architecture and roles in the case of toxic and pathological conditions [12].

Gallic Acid

Gallic acid (GA), a 3,4,5-trihydroxy benzoic acid found in flower petals and fruit peels, has a variety of positive health effects, consisting of anti-inflammatory, antioxidant, neuroprotective, anti-cancer, analgesic, and anti-diabetic characteristics [13]. Gallic acid is a natural compound classified as a trihydroxybenzoic acid and is considered a type of phenolic acid. It is categorized as an organic acid. This compound is naturally occurring and can be found in various plant sources such as gallnuts, sumac, witch hazel, tea leaves, and oak bark. In addition to its presence in nature, gallic acid has applications in the pharmaceutical industry.

One notable attribute of gallic acid is its demonstrated cytotoxicity against cancer cells while sparing healthy cells. This property has garnered attention for its potential use in cancer treatment and research. Moreover, gallic acid is employed as a remote astringent in cases of internal hemorrhage, where it helps to contract and constrict blood vessels to control bleeding. Additionally, it has been utilized in the treatment of albuminuria and diabetes, indicating its potential therapeutic benefits in managing certain medical conditions.

In summary, gallic acid is a versatile compound found in various plants, with applications in pharmaceuticals and promising attributes such as its cytotoxicity against cancer cells and its use in managing internal hemorrhage, albuminuria, and diabetes [14].

Saponins

Plants contain some secondary metabolites saponins are one of them [15]. They have a particular capacity to create pores in the membrane. Saponins have a lytic effect on the membranes of erythrocytes [16]. Saponins act as an insecticidal compound as it is toxic to many insects, pests of crops, and stored grains [17]. The pharmacological properties of saponins include anti-tumor, anti-oxidative, anti-inflammatory, antidiabetic, and neuro-protective actions it also reduces plasma cholesterol level, and cardioprotective activity [18].

Tannins

Tannin has antimicrobial properties that include inhibiting extracellular microbial enzymes, depriving microbes of the nutrients they need to develop, or directly affecting their metabolism by preventing oxidative phosphorylation [19]. Through a variety of methods, including iron chelation, suppression of cell wall construction, rupture of the cell membrane, and blockage of the biosynthetic routes for fatty acids, tannins have been shown to inhibit the development of bacteria. Tannins can block quorum sensing and reduce the gene expression of several virulence factors, including biofilms, enzymes, adhesins, motility, and toxins. Moreover, hydrogels and nanoparticles containing tannin have potent antibacterial properties [20].

Medicinal uses

Antioxidant Activity

The ability of pomegranate juice to act as an antioxidant was evaluated using four different methods: The effects of red wine and green tea infusion were contrasted with those of ferric reducing antioxidant power (FRAP) 2,2'-azinobis (3-ethylbenzothiazoline-6-sulfonic acid) diammonium salt radical cation (ABTS), and 2,2'-diphenyl-1-picrylhydrazyl (DPPH). Commercial Punica granatum juice has three times the antioxidant activity of red wine and green tea put together. Test juices that were only found in arils were less potent than commercial fluids derived from the whole p. granatum. Punicalagin, a pomegranate tannin, is present in commercial juices, however, studies using high-performance liquid chromatography-mass spectrometry (HPLC-MS) and high-performance liquid chromatography with diode-array detection (HPLC-DAD) found that the experimental liquid made from lab arils had just traces of this substance. This could lead to an increase in antioxidant activity [21].

Antibacterial Activity

Antimicrobial is a material that kills or hinders the growth of microorganisms such as protozoans, bacteria, fungi, etc. Based on the mechanism of action, antimicrobials are divided into two wide categories: Microbicidal disinfectants without leaving any mechanism of action and survival, and microbistatics stop all metabolic activities of vital bacteria that survive, called growth inhibitors germs. Pomegranate peel has been found to exhibit antibacterial and antioxidant effects in vitro model systems. It is said that every component of pomegranate peels has medicinal benefits. Pomegranate peel extracts are antibacterial against Staphylococcus aureus, Pseudomonas aeruginosa, and E. coli bacterial pathogens. Pomegranate fruit pericarp was procured. The peels were then divided into smaller pieces and rinsed twice: once with tap water and once with distilled water. After that, it dried outside in the sun until all the water vapor vanished. After that, the pericarp was placed in a hot air oven for three to four days to dry. Then, the dried pericarp was taken for grinding with a mixer grinder. The plant sample was then used throughout the trial in powdered form [22].

Cardioprotective Property

Through the suppression of low-density lipoproteins (LDL) oxidation and vessel relaxation, proanthocyanidins alleviate cardiovascular disorders. Proanthocyanidins can prevent the lectin-like oxidized LDL receptor-1 (LOX-1) from recognizing oxidized low-density lipoprotein LDL, which is a factor in the pathophysiology of arteriosclerosis. Numerous proanthocyanidins have a role in the release of endothelial no and subsequent rise in cyclic guanosine monophosphate (cGMP) levels in incGMPular smooth muscle cells, both of which are endothelium-dependent relaxing activities. Procyanidins alter the no/cyclic GMP pathway, protecting the heart. Procyanidin fractions have demonstrated the capacity to reduce LDL oxidation [23].

Anticancer Property

As a result of oxidative stress, reactive oxygen species (ROS) can damage DNA and promote carcinogenesis in cells. Phytosterols can protect cells against ROS-induced damage, as demonstrated by the fact that sitosterol boosted the activity of antioxidant enzymes in macrophage cells under oxidative stress caused by phorbol 12-myristate 13-acetate. Potentially, -sitosterol prevented the growth of both good and bad estrogen receptor-containing cells. It was shown that activating serine palmitoyl transferase activity, which in turn triggered de novo ceramide formation, was one of the mechanisms behind this impact. It has also been suggested that phytosterols exert their anticancer properties by promoting cell cycle arrest, inducing apoptosis, and enhancing sphingomyelin turnover. MDA-MB-231 cell lines and the MAP kinase and mitogen-activated protein kinase pathways. It demonstrates that the treatment with phytosterols reduces cell proliferation by up-regulating cholesterol synthesis from mevalonate and stimulating the mitogen-activated protein kinase (MAPK) pathway [24].

Wound Healing Property

A wound might be characterized as an epithelial fracture with compromised skin integrity, a loss of anatomic and cellular integrity, or a loss of functional continuity in a live organism. Lesions are physical wounds that result in opening or bleeding. They result in losses of epithelium continuity with or without loss of connective tissue beneath the surface. Chronic ulcers may result in the patient's death from multiple organ failure [25,26]. Wounds are classified into four major types open wound, close wound, acute wound, and chronic wound.

Lesions are categorized as open or closed based on what initially caused the wound. There are also distinctions made between acute and long-term ulcers in terms of their duration and healing process.

Open wound: The wounds do not heal in the open wound. Blood is drained from the body in this condition, and bleeding is evident. It is also described as a cut wound, a wound that does not heal, and such a wound as external bruises or cuts, piercing wounds, penetration wounds, bruises, or bullet wounds [27].

Closed wound: Blood escapes the circulatory system but remains in the body in closed wounds. Contusions or bruises, hematomas or blood tumors, and crush injuries are all examples [27].

Acute wound: An acute lesion is a tissue injury that usually follows an ordered & fast healing process that results in long-term functional and anatomic repair integrity. Cuts or punctures are the most common causes of acute wounds. Full wound healing and surgical incision within the period anticipated [27].

Chronic wound: Incurable wounds are unable to continue with the usual stages of healing so get into pathological inflammatory conditions or chronic wounds that take longer to recover or to replicate regularly. Hypoxia, foreign bodies, local infection, trauma as well general problems such as diabetes mellitus, immunity, or medication are the most common reasons for chronic ulcers [27].

Wound healing is a complex and dynamic process that begins in response to injury, whether from a lesion, surgery, or trauma. It unfolds in three distinct stages, ultimately culminating in tissue repair. The initial phase, known as the inflammatory stage, involves the body's response to the injury, triggering the release of immune cells and proteins to control infection and clear debris. Following this, the fibroblastic phase commences, where specialized cells called fibroblasts generate new collagen and tissue components to bridge the wound. Lastly, the remodeling phase occurs, during which the newly formed tissue matures and strengthens, gradually restoring the wound's structural integrity. These sequential stages ensure the wound undergoes a well-orchestrated healing process, leading to its final repair [27].

The proliferation phase prepares the healing area and prevents the lesion by leading it to expand again and be sore, so that movement can be limited. The fibroblastic phase restores the structure, then the repair phase provides the final result [28].

Inflammatory phase: The proliferation phase or inflammatory phase begins following surgery, or injury and lasts for 24 and 48 hours, with some cases lasting up to two weeks. The inflammation section regulates the hemostatic procedures. To terminate oozing in the lesion region as soon as possible. Inflammation is a significant clinical indicator. The terms rubor, calorie, tumor, dolor, and function-less are used interchangeably. The result of this section is Blood transfusion causes vasoconstriction and platelet aggregation again constipation and vasodilation. To cause inflammation in the wound, phagocytosis is used [29].

Fibroblastic phase: The succeeding stage of wound healing is fibroblastic. The phase lasts two to three days after the surgery stage of inflammation. This section has three steps that are: - granulation, contraction, and epithelialization.

New blood vessels are created during the granulation stage by fibroblasts forming a collagen bed once again. Collagen and glycosaminoglycans, among other substances produced by fibroblast, are crucial for lesion healing. To eliminate faults in the third stage of epithelial tissue that was created on the surface of the wound site, pull together the lesion margins as you approach them [28].

Remodeling phase: This stage might last anywhere from three weeks to two years. At this point, new collagen begins forming. The intermolecular cross-linking of collagen with other proteins promotes tissue strength through vitamin C-based hydroxylation. The scar is also less visible. Red tissue becomes 80% stronger than the original.

The wound-healing properties of plants since taking a look in myths. A large number of ayurvedic herbal plants are crucial to the healing of wounds. Plants are powerful cures because they promote disease to fix the ways naturally. Large-scale research was done instead of healing the wounds treated with medicinal plants. Herbal medicine lesion management includes debridement, sterilization, and providing a humid habitat to promote the setup of an appropriate natural habitat healing process [28].

Periodontal Implications of Pomegranate

Inhibition of matrix metalloproteinases (MMPs) production and IL-1-induced tissue damage is a common property of pomegranate fruit extract. In addition to the aforementioned methods, pomegranate's anti-inflammatory effects may also be a result of its immunoregulatory effects on macrophages, T lymphocytes, and B lymphocytes [30].

Other uses

Skin Whitening

One of the fruits or vegetables with the greatest ellagic acid (EA) concentration is the pomegranate. EA, a phenolic chemical, is used to shield skin from oxidative damage. Since tyrosinase enzymes, which are the primary enzymes catalyzing the formation of melanin, include copper ions, EA is now permitted as a lightening agent for cosmetic products [31].

Recurrent Aphthous Stomatitis

Recurrent aphthous stomatitis (RAS) is a widespread inflammation that has no known etiology. RAS has been connected to a variety of risk and predisposing factors. The RAS procedure has several facets. Genetic predisposition, hematologic abnormalities, microbiological or immunologic variables, trauma, stress, and hormonal state are the main risk factors for this illness. Punica granatum is a fantastic treatment for minor aphthous ulcers. The advantages of using PG in RAS treatment include clinical improvement, which includes pain relief and a faster recovery time, patient compliance, ease of use, and few side effects [32].

Anti-proliferation Effects

Punica granatum roots contain flavonoids that target kinases that phosphorylate proteins at particular locations. Four signaling pathways that flavonoids interact with include protein kinase B (Akt/PKB), mitogen-activated protein kinase (MAP), phosphoinositide 3-kinase (PI3-kinase), and tyrosine kinase P1KC (protein-1 kinase C). Flavonoids' stimulatory and inhibitory effects on these pathways affect how cells function by altering the phosphorylation of the target molecules and by affecting gene expression. Flavonoids can alter growth signaling by inhibiting receptor phosphorylation or impeding growth factor receptor binding. Additionally, flavonoids inhibit the activity of lymphocyte-specific protein tyrosine kinase (Lck) and proto-oncogene tyrosine-protein kinase (Fyn), two notable members of the SRC family of nonreceptor kinases implicated in signaling in the transport of T cells. The ability to interact with the proteins involved in signaling cascades is still present in flavonoids' metabolites. The involvement of flavonoids in cell signaling may be one of the reasons for their anti-proliferative effects [33].

Alteration of Microbiota in Infants

Ellagitannins (ETs) from pomegranate juice (PomJ) are converted to ellagic acid during metabolism (EA). EA is further transformed by intestinal bacteria into urolithins, which are taken into the bloodstream and may have health advantages. PomJ intake during pregnancy has been linked to baby neuroprotection. Nursing infants receive EA and metabolites from their moms who consume PomJ through their breast milk. Breast milk lactococcus, subdoligranulum, and acinetobacter abundance dramatically decreased after PomJ ingestion, however, firmicutes/fecal bacterium abundance significantly increased. Escherichia/Shigella was inversely linked with urolithin a-glucuronide in breast milk (UAG). Lachnoclostridium and Staphylococcus were more prevalent in newborn feces. Infant stool blautia and mother plasm urolithin B-glucuronide were positively associated (UBG). EA and its metabolites are eliminated in the urine, absorbed from breast milk by nursing infants, and affect the infant's gut microbiota. Over time, the amount of EA metabolites in breast milk increased [34].

## Conclusions

The pomegranate plant has a long history of usage as a source of natural medicines and herbal cures. All around the world, medicine's function has progressively expanded. It is currently standard practice to produce medications and nutraceuticals using a plant's component or a plant's chemical compounds. This review intends to shed light on the Punica granatum plant's therapeutic properties as well as the progression of folk medicines into contemporary medicine. Punica granatum has strong antioxidant properties. Punicic acid, alkaloids, ellagitannins, sucrose, anthocyanins, fructose, light organic acids, and other minerals are abundant in this fruit, which also possesses anti-inflammatory and antihypertensive characteristics. Numerous cancers, rheumatoid arthritis, cardiovascular conditions, osteoarthritis, and other disorders can be prevented and treated using Punica granatum. It also aids in the healing of wounds and is healthy for the reproductive system. Punica granatum can attract its beneficial benefits by determining how many soluble nutrients it contains and how it is digested in its genetic makeup. Additional clinical trials are required to fully grasp the restorative potential of Punica granatum, even though several in vitro, animal, and clinical experiments have been carried out to assess and confirm the remedial benefits of these substances.
